# Patients With Short Bowel on Narcotics During 2 Randomized Trials Have Abdominal Complaints Independent of Teduglutide

**DOI:** 10.1177/0148607116663481

**Published:** 2016-08-09

**Authors:** Ken Fujioka, Khursheed Jeejeebhoy, Ulrich-Frank Pape, Benjamin Li, Nader N. Youssef, Stéphane M. Schneider

**Affiliations:** 1 Department of Diabetes and Endocrinology, Scripps Clinic, San Diego, California, USA; 2 Department of Medicine, St. Michael’s Hospital, Toronto, Ontario, Canada; 3 Department of Hepatology & Gastroenterology, Charité University Medicine, Berlin, Germany; 4 Department of Statistics, NPS Pharmaceuticals, Inc., Lexington, Massachusetts, USA; 5 Clinical Research and Development, NPS Pharmaceuticals, Inc., Lexington, Massachusetts, USA; 6 Gastroenterology and Clinical Nutrition, University of Nice-Sophia Antipolis, Nice, France; * Amicus Therapeutics, Cranbury, New Jersey, USA, for BL and Digestive Healthcare Center, Hillsborough, New Jersey, USA, for NNY

**Keywords:** intestinal failure, peristalsis inhibition, intestinal absorption

## Abstract

*Background:* Narcotic agents are frequently administered to manage increased intestinal motility in patients with short bowel syndrome, but long-term use is associated with gastrointestinal (GI) complaints. This analysis evaluated the incidence of narcotic use and abdominal adverse events among patients with short bowel syndrome receiving teduglutide. *Materials and Methods:* Pooled data from patients who received ≥1 dose of teduglutide 0.05 mg/kg/d (n = 77) or placebo (n = 59) in either of 2 randomized, double-blind, phase III studies were analyzed. *Results:* Of 136 patients, 52 (38%) received narcotics. GI adverse events occurred more often among patients who received narcotics than among those who did not (abdominal pain, 51% vs 21%; nausea, 42% vs 11%; abdominal distension, 17% vs 8%; vomiting, 19% vs 6%). Logistic regression analysis indicated that the probability of GI adverse events was significantly increased in patients with narcotic use (*P* = .0009). In contrast, teduglutide treatment, as well as the interaction between teduglutide and narcotic use, did not affect the probability of GI adverse events. *Conclusions:* These results suggest that patients with short bowel syndrome receiving narcotics have chronic GI complaints independent of teduglutide treatment. Data included in this analysis were derived from ClinicalTrials.gov NCT00081458 and NCT00798967 (EudraCT 2004-000438-35 and 2008-006193-15).

## Clinical Relevancy Statement

Adverse gastrointestinal effects are a common complaint among patients with short bowel syndrome receiving narcotic agents as well as those receiving teduglutide, making it unclear the extent to which these complaints are attributable to narcotic agents or teduglutide. This post hoc analysis of pooled data from 2 randomized, double-blind, phase III studies suggests that patients with short bowel syndrome receiving narcotics have chronic complaints independent of teduglutide treatment. These findings are clinically relevant and suggest the importance of careful monitoring and possible dose adjustment of coadministered oral agents during teduglutide therapy.

## Introduction

Narcotic agents, which are often used to manage increased intestinal motility in patients with short bowel syndrome (SBS),^1^ may be associated with gastrointestinal (GI) complaints, including abdominal pain, constipation, bloating, nausea, and vomiting.^2,3^ GI complaints also have been recorded as common adverse events (AEs) during phase III clinical trials of teduglutide in patients with SBS who were dependent on parenteral support (PS; parenteral nutrition [PN] and/or intravenous [IV] fluids).^4–8^

These treatment-emergent GI AEs were not unexpected; they are consistent with both the underlying condition of SBS^9^ and with the known intestinotrophic mechanism of action of teduglutide, a glucagon-like peptide 2 (GLP-2) analogue that promotes intestinal adaptation and intestinal growth and increases absorptive capacity in patients with intestinal failure associated with SBS.^10^ However, because GI complaints were recorded in patients randomized to active drug and those who received matching placebo,^5,7^ it is not clear to what extent they were attributable to teduglutide or narcotic agents, especially considering that a large number of patients in both treatment groups were receiving narcotic agents. This post hoc analysis was undertaken to explore the correlations between the incidence of narcotic use and abdominal complaints reported by patients with SBS during the phase III clinical studies of teduglutide, with the aim of understanding better the possible origins of these GI symptoms.

## Methods

Pooled data were analyzed from patients who received at least 1 dose of subcutaneous teduglutide at the US Food and Drug Administration (FDA)– and European Medicines Agency (EMA)–approved dose^7,11^ of 0.05 mg/kg/d or placebo in 2 randomized, double-blind, 24-week, phase III clinical trials, the pivotal STEPS study (ClinicalTrials.gov, NCT00798967; EudraCT, 2008-006193-15)^5^ and study NCT00081458 (EudraCT, 2004-000438-35).^4^ Although a higher teduglutide dose, 0.10 mg/kg/d, was evaluated in NCT00081458,^4^ data from patients in that treatment arm were not included in this post hoc analysis, which focused on the FDA- and EMA-approved dose of 0.05 mg/kg/d. In both studies, patients, investigators, and other personnel related to the study were blinded to the treatment assignment; teduglutide and placebo were identical in appearance.

Both studies enrolled adult patients with SBS resulting from major intestinal resection secondary to vascular ischemic disease, volvulus, cancer, Crohn’s disease, or injury. Patients with a history of Crohn’s disease were required to have been in clinical remission for at least 12 weeks before dosing. All patients were required to have been dependent on PS for at least 12 months and to be receiving infusions at least 3 times per week for fluids, electrolytes, or nutrients.

Major exclusion criteria included active inflammatory bowel disease, history of significant systemic diseases (eg, cardiovascular, respiratory, renal, infectious, endocrine, hepatic, or central nervous system), or radiation enteritis; prior use of native GLP-2 or teduglutide; use of growth hormone or growth factors within the past 3 months (NCT00081458) or 6 months (NCT00798967 [STEPS]); cancer within the past 5 years (not including resected cutaneous basal or squamous cell carcinoma or in situ nonaggressive and surgically resected cancer); and premalignant or malignant change in colonoscopy biopsy or polypectomy (untreated condition for NCT00798967 [STEPS]; any condition for NCT00081458).

Local institutional review boards or medical ethics committees approved the protocols for both studies, which were conducted in accordance with applicable International Conference on Harmonization Good Clinical Practice guidelines and the World Medical Association Declaration of Helsinki and its amendments concerning medical research in humans. All study patients provided informed consent.

For this statistical analysis, data were pooled and analyzed using descriptive statistics and logistic regression. The dependent variable of GI complaints was identified by treatment-emergent AEs of abdominal pain, nausea, abdominal distension, vomiting, constipation, intestinal obstruction, appetite disorders, and GI stenosis and obstruction. Independent variables were teduglutide treatment, narcotic use, and the interaction between teduglutide treatment and narcotic use. Duration of narcotic exposure was not included as a parameter (narcotic usage captured as a binary yes/no response). Narcotic agents assessed in this analysis were those captured in the concomitant medication record and included opium alkaloids, derivatives, and expectorants; opioid anesthetics; other opioids; drugs used in opioid dependence; benzomorphan derivatives; diphenylpropylamine derivatives; and phenylpiperidine derivatives. Loperamide was classified as an antipropulsive agent and was not included in this analysis.

## Results

During the 2 randomized controlled studies included in this exploratory analysis, 77 patients received teduglutide 0.05 mg/kg/d and 59 patients received placebo. Patient demographics and baseline characteristics, which have been published previously, were not different between treatment groups in either of the 2 studies.^4,5^

Sixty-six patients (86%) in the teduglutide group and 54 patients (92%) in the placebo group completed 24 weeks of treatment. Eleven patients (14%) in the teduglutide group discontinued the study early, 8 because of AEs, 2 because of withdrawal of consent, and 1 because of a randomization error. In the placebo group, 5 patients (8%) discontinued early, 4 because of AEs and 1 because of withdrawal of consent. Six teduglutide-treated patients (8%) discontinued treatment because of GI-related complaints, including abdominal distension (n = 2), constipation (n = 2), abdominal pain (n = 1), nausea (n = 1), vomiting (n = 1), and hemorrhoidal hemorrhage (n = 1; patients could report more than 1 AE). In the placebo group, 3 patients (5%) discontinued early because of GI-related complaints of increased fecal volume (n = 1), frequent bowel movements (n = 1), and intestinal polyp (n = 1). Overall, GI-related treatment-emergent AEs were common in both the teduglutide-treated and placebo-treated groups; a higher percentage of patients receiving teduglutide experienced abdominal complaints compared with patients receiving placebo (Table 1).

**Table 1.** Treatment-Emergent Gastrointestinal Complaints Associated With Study Drug and/or Narcotic Agents.^a^

	Teduglutide 0.05 mg/kg/d (n = 77), No. (%)			Placebo (n = 59), No. (%)		
Adverse Event	All Teduglutide (n = 77)	Narcotics^b^ (n = 32)	No Narcotics (n = 45)	All Placebo (n = 59)	Narcotics^b^ (n = 20)	No Narcotics (n = 39)
Abdominal pain	29 (38)	18 (56)	11 (24)	16 (27)	9 (45)	7 (18)
Nausea	19 (25)	16 (50)	3 (7)	12 (20)	6 (30)	6 (15)
Abdominal distension	15 (19)	9 (28)	6 (13)	1 (2)	0 (0)	1 (3)
Vomiting	9 (12)	7 (22)	2 (4)	6 (10)	3 (15)	3 (8)

a Pooled data for patients who received ≥1 dose of double-blind study drug (teduglutide 0.05 mg/kg/d or matching placebo) in NCT00081458 (EudraCT, 2004-000438-35) or STEPS (ClinicalTrials.gov, NCT00798967; EudraCT, 2008-006193-15) studies; abdominal complaints based on Medical Dictionary for Regulatory Activities terms.

b Concomitant narcotic agents (eg, opium alkaloids and derivatives, opioid analgesics, or other opioids) in the teduglutide and placebo groups, respectively, included, in order of frequency the following: codeine (n = 14, 18%; n = 4, 7%), tramadol (7, 9%; 1, 2%), hydromorphone (5, 7%; 1, 2%), morphine (3, 4%; 3, 5%), oxycodone/acetaminophen (3, 4%; 2, 3%), codeine/paracetamol (Panadeine) (3, 4%; 1, 2%), hydrocodone/acetaminophen (Vicodin) (2, 3%; 1, 2%), dextropropoxyphene hydrochloride/paracetamol (Di-Gesic) (2, 3%; 0, 0%), oxycodone (1, 1%; 4, 7%), galenic/paracetamol/codeine (1, 1%; 1, 2%), acetaminophen/codeine (Procet) (1, 1%; 1, 2%), acetaminophen/propoxyphene (Propacet) (1, 1%; 1, 2%), dextromethorphan hydrobromide/guaifenesin (Tussin DM) (1, 1%; 1, 2%), dextropropoxyphene hydrochloride/acetaminophen (Aporex) (1, 1%; 0, 0%), sulfamethoxazole trimethoprim (Bactrizol) (1, 1%; 0, 0%), dextromethorphan (1, 1%; 0, 0%), fentanyl (1, 1%; 0, 0%), acetaminophen/salicylamide (Frenadol) (1, 1%; 0, 0%), paracetamol/dihydrocodeine tartrate (Remedeine) (1, 1%; 0, 0%), tramadol/acetaminophen (Ultracet) (1, 1%; 0, 0%), hydrocodone bitartrate/guaifenesin (Codiclear) (0, 0%; 1, 2%), hydrocodone (0, 0%; 1, 2%), methadone (0, 0%; 1, 2%), nicomorphine (0, 0%; 1, 2%), oxycodone/acetaminophen (Oxycocet) (0, 0%; 1, 2%), and noscapine/promethazine (Tussisedal) (0, 0%; 1, 2%). Patients may have received >1 narcotic agent.

**Figure 1.**
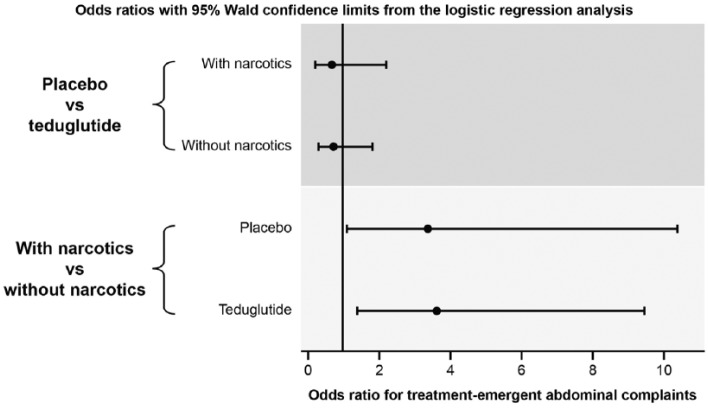
Correlation of treatment-emergent gastrointestinal complaints with concomitant narcotic use. Pooled data for patients who received ≥1 dose of teduglutide 0.05 mg/kg/d or matching placebo. Upper graph, probability of abdominal adverse events was not significantly different between treatment groups (95% CIs include 1). Lower graph, narcotic use related to a higher adverse event rate (95% CI lower limits >1).

Fifty-two of 136 (38%) patients enrolled in the 2 studies received narcotic agents at some point during the 24-week study periods. Thirty-two of 77 (42%) patients randomized to teduglutide and 20 of 59 (34%) randomized to placebo received at least 1 concomitant narcotic agent (for list, see Table 1 footnote). Overall, abdominal pain was more frequent among patients who received narcotics (27/52; 52%) than among those who did not (18/84; 21%). A similar pattern was observed for GI AEs of nausea (42% vs 11%, respectively), abdominal distension (17% vs 8%), and vomiting (19% vs 6%). The incidence of abdominal pain, nausea, and vomiting was higher among patients receiving narcotics, regardless of whether patients had been assigned to the teduglutide or placebo treatment group (Table 1). When the data were analyzed in a logistic regression model, the probability of GI AEs (including abdominal pain, nausea, abdominal distension, vomiting, constipation, intestinal obstruction, appetite disorders, and GI stenosis and obstruction) was significantly increased in patients with narcotic use, regardless of treatment group (*P* = .0009; data not shown). Furthermore, the interaction between treatment and narcotic use did not change the likelihood of GI AEs (data not shown). Figure 1 (upper graph) illustrates that no significant correlation was detected between treatment groups and probability of GI AEs, whereas narcotic use was related to a higher AE rate (lower graph).

## Discussion

Pooled data from the teduglutide placebo-controlled studies indicate that a large proportion of patients received concomitant narcotics at some point during the 24-week study periods (42% of patients in the teduglutide arm and 34% of patients in the placebo arm), reflecting current practice for the symptomatic management of SBS. Within both groups, most GI AEs were more common among patients who received narcotics (Table 1). The finding that a higher percentage of patients who received narcotics (compared with those who did not) reported events of abdominal pain, nausea, and vomiting suggests that, independent of teduglutide, chronic GI complaints in patients with SBS receiving narcotics are a major symptom management challenge. Regression analysis supported the findings and showed that narcotic use significantly increased the probability of GI AEs in both the teduglutide and placebo study arms.

The major limitation of this report is that it is a post hoc analysis in what is, by necessity in this rare disease state, a relatively small number of patients. Furthermore, the assessed narcotic agent data were limited to the details captured in the concomitant medication record. The scope of interpretation of these findings should be curtailed because of the lack of analysis determining whether a specific GI symptom is attributable to the underlying disease state or some combination of treatment-related factors. In addition, the temporal relationship between the initiation of the concomitant narcotic agent and the onset of GI AEs was not determined in this analysis. GI AE onset could have been separated from initial administration of a narcotic drug. Similarly, rather than preceding a GI event, narcotic agents could have been administered in response to a GI AE. Nonetheless, the current analysis indicates that narcotic use is associated with an increased probability of a GI AE in patients with SBS.

One of the precautions for the use of teduglutide is that it has the potential to increase absorption of concomitant oral medications.^7^ It was beyond the scope of this analysis to determine whether early reduction of narcotic dosage might have prevented possible GI AEs, particularly abdominal pain. These findings suggest the importance of careful monitoring and possible dose adjustment of coadministered oral agents—including narcotic agents—during teduglutide therapy.
